# Optimising personal continuity: a survey of GPs’ and older patients’ views

**DOI:** 10.3399/BJGPO.2022.0099

**Published:** 2023-03-22

**Authors:** Lex Groot, Marije te Winkel, Henk Schers, Jako Burgers, Martin Smalbrugge, Annemarie Uijen, Henriëtte van der Horst, Otto Maarsingh

**Affiliations:** 1 Department of General Practice, Amsterdam UMC, Vrije Universiteit Amsterdam, Aging and Later Life, Amsterdam Public Health, Amsterdam, The Netherlands; 2 Department of Primary and Community Care, Radboud University Nijmegen Medical Centre, Nijmegen, The Netherlands; 3 Maastricht University Medical Center+, Maastricht University, Department of General Practice, Care and Public Health Research Institute (CAPHRI), Maastricht, The Netherlands; 4 Amsterdam UMC, Vrije Universiteit Amsterdam, Department of Medicine for Older People, Boelelaan, Amsterdam, The Netherlands

**Keywords:** aged, aged, 80 and over, continuity of care, general practice, Netherlands, physician-patient relations, quality of life, surveys and questionnaires

## Abstract

**Background:**

Personal continuity — having a GP who knows their patients and keeps track of them — is an important dimension of continuity of care and is associated with lower mortality rates, higher quality of life, and reduced healthcare costs. In recent decades it has become more challenging for GPs to provide personal continuity owing to changes in society and health care.

**Aim:**

To investigate GPs’ and older patients’ views on personal continuity and how personal continuity can be improved.

**Design & setting:**

Cross sectional survey study in The Netherlands.

**Method:**

A digital and postal survey was sent to 499 GPs and 1599 patients aged 65 years or older. Results were analysed using descriptive statistics for quantitative data and thematic analysis for open questions.

**Results:**

In total, 249 GPs and 582 patients completed the surveys. A large majority of GPs (92–99%) and patients (91–98%) felt it was important for patients to see their own GP for life events or psychosocial issues. GPs and patients provided suggestions on how personal continuity can be improved. The thematic analysis of these suggestions identified nine themes: 1) personal connection, 2) GP accessibility and availability, 3) communication about (dis)continuity, 4) GP responsibility, 5) triage, 6) time for the patient, 7) actions by third parties, 8) team continuity, and 9) GP vocational training.

**Conclusion:**

Both GPs and older patients still place high value on personal continuity in the context of a changing society. GPs and patients provided a wide range of suggestions for improving personal continuity. The authors will use these suggestions to develop interventions for optimising personal continuity in general practice.

## How this fits in

Personal continuity — having a GP who knows their patients and keeps track of them — is a core value of general practice and is associated with many benefits, especially for older patients. Due to changes in society and health care it has become more difficult for GPs to provide personal continuity. This study shows that GPs and older patients still place high value on personal continuity. Furthermore, this study provides a wide range of suggestions to improve personal continuity. The authors will use these suggestions to develop interventions for optimising personal continuity.

## Introduction

Personal continuity is a core value of general practice and is highly valued by both patients and GPs.^1–7^ It involves a GP who knows their patients and keeps track of them in different healthcare settings and in the course of time.^8–10^ Personal continuity is associated with many benefits, such as higher quality of GP care,^11–14^ higher quality of life,^15–17^ reduced healthcare costs,^18–20^ and lower mortality rates.^19–26^

Due to changes in society and health care, it has become challenging for GPs to provide personal continuity. GPs increasingly work part-time, organise themselves in larger practices, and often work as a locum GP.^27–29^ At the same time, the need for personal continuity is increasing owing to higher numbers of older patients and patients with multiple chronic conditions. These patients are known to benefit the most from personal continuity, yet are more at risk for receiving fragmented care.^7,30–35^

In addition, in some countries (for example, Belgium and Norway) government policies facilitate GPs in providing personal continuity, whereas in other counties (for example, the Netherlands and the UK) policies may prioritise accessibility over personal continuity.^8,36–38^

Previous research has demonstrated that personal continuity is viewed as important by both GPs and patients.^1,2^ However, healthcare systems may have gradually become more oriented toward principles other than personal continuity.^38,39^ Therefore, it is possible that GPs and older patients have come to put less value on personal continuity.

Little is published or disseminated on how personal continuity could be improved.^26,40–42^ As it has become more challenging for GPs to provide personal continuity, strategies for improving personal continuity are necessary.

The aim of this study was to investigate GPs’ and older patients’ views on personal continuity and how it can be improved. This study is part of a larger study aiming to develop and evaluate a multicomponent intervention for optimising personal continuity in general practice (Netherlands Trial Register, trial NL8132).^43^

## Method

### Setting

In 2019, 12 766 GPs were registered in The Netherlands, working in group practices (36%), two-handed practices (32%), single-handed practices (14%), or as a locum (18%).^29^ Dutch general practices employ practice assistants who are responsible for planning consultations, telephone triage, and performing supportive medical tasks. Practices also often employ practice nurses to support the GP in care for chronic diseases, such as diabetes, pulmonary conditions, or mental health issues.^44^

### Participants and data collection

#### GPs

In April and May 2019, eight regional GP networks from the west (*n* = 4), centre (*n* = 2), east (*n* = 1), and south (*n* = 1) of The Netherlands were contacted for the distribution of a web-based GP survey. These networks included primary care practice-based research networks and local collaborations of GP practices. Using these networks, 499 GPs were invited to participate by sending an invitation and hyperlink via email. The survey was distributed using Survalyzer, an online program for developing and distributing surveys.^45^

As the response rate to surveys is known to be limited, virtual snowball sampling was used to increase the number of responders.^46^ The 499 invited GPs were encouraged to share the link to the digital survey with other GPs and asked to complete the survey within 2 weeks. No reminder was sent.

#### Patients

Between May and August 2019, practices from the west (*n* = 7), centre (*n* = 8) and east (*n* = 2) of the Netherlands were invited for the study to include patients aged 65 or older. Patients aged 65 or older were focused on as these patients benefit the most from personal continuity and may be more impacted by fragmentation of care.^7,16,35^ The consenting GPs were instructed to select a random sample of 30–50 eligible patients from their electronic medical record, depending on their practice size and the number of eligible patients. The eligibility criteria for patients were: aged 65 years or older, registered in the practice for at least 1 year, living at home, with no severe cognitive disabilities, and able to understand and write Dutch. GPs could exclude patients for any reason; for example, terminal illness.

The practices were selected to constitute a purposeful sample with variation in practice size, number of GPs employed, and level of urbanisation.

In total, 1599 patients were included. These patients received the questionnaire from their GP by post, accompanied by a letter of recommendation. No reminder was sent.

#### Questionnaires

The GP survey (14 questions, Supplementary File S1) and the patient survey (20 questions, Supplementary File S2) consisted of three parts.

##### 1. Participant characteristics

Relevant personal and practice characteristics were collected from all participants. For GPs this included sex, employment status, working experience, time in clinical activities per week, number of GPs employed in practice, urbanisation of practice area, and number of patients registered at the practice. For patients, it included sex, age, period registered at practice, living situation, nationality, number of GP contacts in the past 12 months (including telephone calls and home visits), number of chronic diseases, and experienced disability.

##### 2. Views on personal continuity

GPs and patients were presented with nine scenarios and were asked how important they felt it was to see their own patients or own GP on a 5-point or 3-point scale, respectively (Box 1). All scenarios and questions were adopted verbatim from Schers *et al*.^1,2^ A brief summary of the survey studies by Schers *et al* is displayed in Table 1

Box 1 **Scenarios adopted from Schers *et al* ^1,2^ presented to patients and GPs.**

Scenario

1. Splinter in the eye
2. Sprained ankle
3. Regular blood pressure check
4. Problems at work
5. Sudden, severe chest pain
6. Unexpected blood in stools
7. Family problems
8. Anxiety about specific abdominal symptoms
9. Discussing future when seriously ill

**Table 1.** Summary of methodological characteristics of the survey studies by Schers *et al* ^1,2^

	GPs’ views on continuity of care	Continuity of care in general practice: a survey of patients’ views
Study aim	To investigate GPs’ views on the importance of personal contact with their own patients, on their responsibility to demonstrate unsolicited concern/empathy, and personal availability outside of office hours.	To explore patients’ anticipated needs for contact with their GP
Setting	Netherlands Institute for Health Research database	35 GP practices throughout The Netherlands
Sampling	Random sample of 500 GPs, no snowballing	25 patients per practice who consecutively visited the practice on a specified day
Inclusion criteria	None	>18 years, GPs could not exclude patients
Survey type	Postal	Postal
Reminder	After 3 weeks	After 2 weeks

GPs and patients were asked whether they perceived a change in personal continuity in the past 5 years on a 5-point Likert scale. They also received a list of previously identified barriers (*n* = 11) and facilitators (*n* = 9) for personal continuity.^3,5,8,9,47–49^ Participants were asked to select items applicable to their perception of change of personal continuity. Participants who perceived no change were asked not to select any items.

##### 3. How can personal continuity be improved?

Both GPs and patients were asked to respond in free text to the question ‘*How would you improve personal continuity in your general practice*?’ to provide suggestions.

### Analysis

The responses were analysed on the Likert scale, multiple choice, and single choice questions by means of descriptive statistics (count, mean, percentages) using SPSS (version 22.0). Five-point Likert scale items were trichotomised for purposes of analysis; that is, the two lowest scoring categories were merged into a single category, as were the two highest . Answers with multiple choices were arranged by frequency of selection to facilitate analysis.

The suggestions for improving personal continuity answered in free text were analysed using thematic analysis.^50^ Here, LG and MW reviewed the data to familiarise themselves with the content. LG and MW individually open coded and sorted the responses based on the content to generate the initial labels. These labels were reviewed and discussed by MW and LG in consecutive meetings to establish the initial themes. As a final step, HS, LG, and MW reviewed the initial themes and adjusted themes where necessary.

## Results

In total, 249 GPs and 582 patients responded to the survey. Patients had a 36% response rate. For GPs, a response rate could not be calculated owing to the use of snowball sampling.^46^ Table 2 shows an overview of responder characteristics.

**Table 2.** Characteristics of survey responders (GPs and patients)

GPs (*n* = 249)		Patients (*n* = 582)	
Characteristic	*n* (%)	Characteristic	*n* (%)
**Sex**		**Sex**	
Male	99 (40)	Male	248 (43)
Female	149 (60)	Female	317 (54)
*Missing*	1 (0)	*Missing*	17 (3)
**Employment status**		**Age, years**	
Partner	185 (74)	65–74	234 (40)
Salaried	31 (12)	75–84	180 (31)
Locum	20 (8)	≥85	57 (10)
Other	13 (5)	*Missing*	111 (19)
**Working experience, years**		**Period registered at practice, years**	
1–10	47 (19)	<1	3 (1)
11–20	103 (41)	1–2	26 (4)
21–30	74 (30)	3–4	50 (9)
>30 years	25 (10)	5–10	77 (13)
		>10	395 (68)
		*Missing*	31 (5)
**Time in clinical activities per week, hours**		**Living situation**	
<16	83 (33)	Alone	138 (24)
16–30	147 (60)	Widowed	76 (13)
31–38	12 (5)	Married/living together	346 (59)
>38	7 (3)	Other	10 (2)
		*Missing*	12 (2)
Number of GPs in employment of practice*		**Nationality**	
1	35 (14)	Dutch	562 (97)
2	95 (38)	Other	8 (1)
≥3	131 (53)	*Missing*	12 (2)
Urbanisation of practice area*		**Number of GP contacts in the past 12 months**	
Very rural	35 (14)	0	33 (6)
Rural	51 (20)	1–2	142 (24)
Average	62 (25)	3–4	179 (31)
Urban	39 (16)	5–10	149 (26)
Very urban	78 (31)	>10	54 (9)
		*Missing*	25 (4)
Number of registered patients*		**Number of chronic diseases**	
<1750	9 (4)	0	253 (43)
1750–1999	12 (5)	1–2	276 (47)
2000–2249	13 (5)	≥3	33 (6)
2250–2500	38 (15)	*Missing*	20 (3)
>2500	177 (71)		
		**Disability due to a chronic disease**	
		Yes	97 (17)
		No	442 (76)
		*Missing*	43 (7)
			

*Practice characteristics on which GPs could give more than one answer.

### Views on personal continuity

The views of GPs and patients on personal continuity are presented in Table 3. Most GPs felt it was important to see their own patients when it concerned life events or psychosocial issues. GPs’ importance for seeing their own patients varied between scenarios, with 9% of GPs percieving it as (very) important to see their own patient for a splinter in the eye, and 99% as (very) important for discussing the future with a seriously ill patient. The majority of patients felt it was important to see their GP in seven out of nine scenarios. Only for a splinter in the eye (30% preferred own GP) or a sprained ankle (45% preferred own GP) did most patients not desire to see their GP.

**Table 3.** Views of GPs and patients on when it is important to have contact with a personal GP

	GPs (*n* = 249)		Patients (*n* = 582)	
*Scenarios*	*Important or very important*			
	*n*	%	*n*	%
Discussing future when seriously ill	248/249	99.6	519/529	98.1
Problems in the family	242/249	97.2	424/460	92.2
Problems at work	230/249	92.4	383/421	91.0
Anxiety about specific abdominal symptoms	222/249	89.2	507/539	94.1
Unexpected blood in stools	161/249	64.7	459/536	85.6
Sudden, severe chest pain	76/249	30.5	427/538	79.4
Regular blood pressure check	47/249	18.9	290/524	55.3
Sprained ankle	25/249	10.0	243/541	44.9
Splinter in the eye	17/249	6.8	162/540	30.0

Over the past 5 years, 54% of GPs (*n* = 135) and 17% of patients (*n* = 98) perceived a decrease in personal continuity, 3% of GPs (*n* = 8) and 24% of patients (*n* = 142) perceived an increase in personal continuity, and 43% of GPs (*n* = 106) and 53% of patients (*n* = 310) perceived no change. All GPs provided a response, whereas 5% of patients (*n* = 32) did not provide a response.

The experienced barriers and facilitators for personal continuity are displayed in Table 4. For GPs, the number of different healthcare providers involved with one patient’s care was perceived as the main barrier for personal continuity (81% of responders), while having a small-scale practice was considered the main facilitating factor (100% of responders). For patients, the main barrier was long waiting times for seeing their own GP (52% of responders). Patients considered putting great value on seeing one’s own GP (76% of responders) to be the main facilitating factor for personal continuity.

**Table 4.** Top five perceived barriers and facilitators for personal continuity in general practice

	GPs who perceived decreased personal continuity (*n* = 135)*		Patients who perceived decreased personal continuity (*n* = 98)*	
		*Response* 135/135		*Response*94/98
Barriers	There are too many healthcare providers involved with one patients' care	81%	It takes too long before I get an appointment with my own GP	52%
	GPs work part-time	59%	My own GP is often not available for me	47%
	Increased scale of primary care (for example, group practices)	51%	My appointments are rarely scheduled with my own GP	34%
	High staff turnover rate	43%	High GP turnover prevents me from making a connection with any GP	28%
	Receptionist schedule patients on the first available spot, rather than at the patients' own GP	36%	I recently changed GPs and do not yet know my current GP	18%
				
	**GPs who perceived increased personal continuity (** ***n*** = 8)*		**Patients who perceived increased personal continuity (** ***n*** = 142)*	
		*Response*8/8		*Response*130/142
Facilitators	Retaining a small-scale practice	100%	I place great value on seeing my own GP	76%
	Low staff turnover rate	75%	I ask for my own GP when making an appointment	72%
	Practice assistants schedule patients with the regular GP, rather than with the first available GP	63%	I will reschedule my agenda in order to get an appointment with my own GP	46%
	Sufficient staff	63%	I have known my own GP for a long time	36%
	Every GP working in the practice has at least 3 days of patient care	63%	My appointments are always scheduled with my own GP	30%
				

* Only participants who perceived change (that is, 143 GPs and 240 patients) were asked to provide information on barriers and facilitators.

### How can personal continuity be improved?

In total, 222 GPs (89%) and 209 patients (36%) provided 316 and 137 suggestions, respectively, for improving personal continuity. No suggestions were provided by 27 GPs (11%) and 373 patients (64%).

Nine themes were identified from GP and patients’ suggestions (Table 5). Patients’ suggestions focused on having a personal connection with their GP, their expectations of GP care, and improving practice accessibility. GPs’ suggestions focused on reforming practice organisation, improving consultation planning, and collaboration with other care organisations such as hospitals.

**Table 5.** Suggestions for improvement of personal continuity by GPs and patients (in no particular order)

Origin		Domains	Quote
*GP*	*Patient*		
		1. Personal connection	
X	X	Organise introductory meetings	P: *‘When I visit my GP, he does not look at me and constantly looks at his screen. My husband and I both had this experience. Is my GP really interested in me and my problem?’*
	X	Improve open consultation and listening skills	
	X	Perform home visits	
	X	Ensure the EMR is up to date and is read by GPs	
	X	Have a personal connection between GP and patient	
		2. GP accessibility and availability	
	X	Implement e-health: consultation by video calls and emails	GP: *‘Personally, I am available to terminal patients directly or via colleagues. If this is not possible due to circumstances, I communicate this with my patient. In my experience, the thought that your expertise is within reach is comforting to these patients.’*
	X	Organise walk-in hours with own GP	
X		Ensure direct GP accessibility and availability outside office hours for own patients with complex needs, particularly palliative care needs	
	X	Reduce waiting times for own GP	
X	X	Reduce part-time employment	
		3. Communication about (dis)continuity	
X	X	Communicate GP availability and staff changed to the patients of the practice	P: *‘I would consider it very pleasant if the website showed the office hours of our GP and the availability, that is, holidays. Preferably a notification well ahead of the absence so that I am not unexpectedly confronted with my GP’s absence when I call my practice for an appointment.’*
X		Encourage patients to ask for their own GP when scheduling appointment	
X		Inform patients about the aim and structure of the out-of-office hours care	
		4. GP responsibility	
X	X	Promote a proactive GP attitude by periodically, or on occasion, initiating contact instead of depending on patient initiative	P: *‘Unfortunately, we do not have a “total body doctor”. However, an annual check-up combined with a positive consultation experience would be appreciated. In particular for older people, just like a technical examination for cars!’*
X		Stimulate GPs to only prescribe recurrent prescriptions for their own patients	
X	X	Instruct assistants to schedule patients with follow-up consultations with same doctor, barring emergency consultations	
		5. Triage dependent on severity and urgency	
X	X	Instruct assistants to schedule complex patients with same doctor, barring emergency consultations	P: *‘The role of the practice assistant is crucial in my opinion. She has to compromise between a (too) busy schedule and the (non)importance of seeing your own GP.’*
X		Optimise the EMR to help assistants with scheduling, that is, preferred GP pop-up	
X		Plan small medical issues or emergencies with locums or nurse physicians to give regular GP more time for complex cases	
		6. Time for the patient	
X		Increase the compensation per consultation	GP: *‘Improve the organisation of out-of-office hours care, in particular with regard to GP with practices. During the day, continuity is essential, emergency care is less continuity-dependent. Enable combining out-of-office hours care with day care, and so create a small workload and provide flexibility.’*
X	X	Reduce administrative burden and work load	
X		Reform organisation of out-of-office hours care	
X	X	Increase the time per consultation	
X		Increase GP availability for direct patient care to at least 3 days a week	
		7. Actions by third parties	
X		Introduce a nationwide EMR	GP: *‘Sufficient transfer of care between inside and outside office care. For example, by having a nationwide EMR [...] In my opinion, the IT should be adapted drastically to the 21st century.’*
X	X	Optimise collaborations with hospitals and mental healthcare organisations	
		8. Team continuity	
X	X	Promote small-scale practices: fewer patients and fewer GPs per practices and stimulate regular employment	GP: *‘In a small-scale practice, personal continuity is better ensured, because the assistants know their patients and the lines of communication are shorter. In a large health centre, I supervised several nurse physicians, medical trainees, GP trainees etc. Although I liked these collaborations, the continuity of care was limited.’*
X	X	Reduce changes of doctors by stimulating regular employment	
X	X	Improve working atmosphere	
X		Increase pay of support staff to improve job attractiveness	
X	X	Ensure a sufficient number of staff	
		9. GP vocational training	
X		Include importance of personal continuity in GP education	GP: *‘[…] More attention in education programmes that being a GP is not ‘just’ a job, that it requires a certain dedication and servitude (like judges). This should be a part of the application procedure.’*

EMR = electronic medical record.

## Discussion

### Summary

This study showed that both GPs and patients aged 65 or older still place high value on personal continuity. The desire for personal continuity is related to the reason for the encounter, where the need for personal continuity increases when facing serious medical conditions and emotional problems. While a majority of GPs perceived a decline in personal continuity, most patients did not perceive any change. A large majority of GPs provided suggestions on how personal continuity could be improved while most patients did not provide any suggestions. The provided suggestions by both GPs and patients covered a broad range of daily practice activities, from improving continuity-centred consultation planning to improving GP availability and accessibility.

### Strengths and limitations

By involving both GPs and patients in this study, it was possible to compare GPs’ and patients’ views on personal continuity and how it can be improved. GP characteristics in the sample closely resemble the characteristics of the overall GP population in The Netherlands, increasing the generalisability of the results.^29^

The majority of patients aged 65 or above in this study had one or more chronic diseases. Such patients are known to benefit the most from personal continuity but are also more at risk for receiving fragmented care.^7,30–35^ Therefore, the suggestions identified in this study may be very relevant to daily practice.

A limitation of the study was the missing data. While GPs’ online responses had no missing data owing to the use of Survalyzer, patients’ postal responses had between 13 (2%) and 81 (14%) missing responses (Supplementary Table S1). At the time, it was decided to use postal surveys for the patients, because GPs usually did not have the email addresses of their randomly selected patients. In addition, it was believed that — compared with an online survey — a conventional postal survey would lead to a higher response rate in this specific older population. Yet, by using postal surveys patients had the opportunity not to answer certain questions, leading to missing data. As the GPs performed the random selection of patients, patients were anonymous to the researchers and it was not possible to approach patients afterwards to complete the surveys or send reminders. However, the impact of non-response bias is considered to be limited, owing to the relatively low rate of missing data, the predominantly qualitative nature of the study, and the sample size.

This study had a relatively low response rate among patients. A low response rate in surveys is often seen as an indicator of study quality. However, while low response rates may influence power and precision, they do not necessarily influence the quality of data.^51^ In addition, response rates are not suitable to judge qualitative data, which comprises the majority of the results.^52^

This study was widely distributed in The Netherlands and involved only Dutch GPs and patients. While this ensures the relevance of the results for general practice in The Netherlands, the findings may not be directly applicable in other healthcare systems.

### Comparison with existing literature

Previous research by Schers *et al* showed that personal continuity was important for GPs and patients.^1,2^ Comparing this study's results to the results of Schers *et al*, GPs' and patients’ views on personal continuity seem to have changed little in the past 20 years (Supplementary Table S2).^1,2^ Compared with 2001, GPs viewed it as less important to provide personal continuity for minor health issues such as a splinter in the eye (from 18% to 9%). Conversely, patients’ need for seeing the own GP for minor health issues remained similar for most situations and increased for a sprained ankle (from 35% to 45%). Patients viewed it as more important to see the own GP for problems at work (from 52% to 91%) or family problems (from 72% to 92%) now compared with 2001. This may suggest that while GPs over time have come to focus their provision of personal continuity more on specific subgroups of patients or health issues, older patients still mostly expect to see the own GP for any ailment.

The present study used the same questions as Schers *et al*.^1,2^ However, differences with regard to aim, sampling, inclusion criteria, survey structure, and sending of reminders between studies may limit the extent to which a direct comparison can be made. Therefore, the above comparison between studies may benefit from further confirmation.

Schers *et al* already concluded that the need for personal care was dependent on the reason for consultation.^1,2^ This was also observed in other studies.^53,54^ The present study has reaffirmed this statement, with very few changes in ranking of the different scenarios used by Schers *et al* in 2002.

Dutch patients are highly satisfied with the quality of GP care overall.^27^ In this study, a majority of patients did not perceive a decrease in personal continuity in the past 5 years. As personal continuity is associated with higher patient satisfaction,^53,55–58^ Dutch patients may already perceive higher levels of personal continuity. This could explain why patients have provided relatively fewer suggestions for improving personal continuity compared with GPs in this study.

Recently, Baker *et al* ^26^ stated that there is a is a high demand for interventions improving personal continuity. The present study contributes to filling this major gap in knowledge by being the first to collect GPs’ and patients’ suggestions on how to improve personal continuity.

### Implications for research and practice

This study generated a broad inventory of suggestions, and an inventory of barriers and facilitators for improving personal continuity. The wide range of suggestions provided in this study can be used in general practice to develop interventions for optimising personal continuity.

While the use of surveys facilitates an explorative approach, data are often not detailed or conceptually rich enough.^59^ Therefore, additional qualitative research is recommended to further develop the suggestions in this articles into strategies for improving personal continuity and to perform a more in-depth analysis of barriers and facilitators.

GPs suggested that changes at policy level are also needed to improve personal continuity; for example, reducing practice size or changing the current reimbursement system. This indicates that there is a role for policymakers in optimising personal continuity in health care.

This study is part of a larger study aiming to develop and evaluate a multicomponent intervention for improving personal continuity in general practice (Netherlands Trial Register, trial NL8132).^43^ The results of this study will be used to further prioritise interventions and determine facilitating factors and barriers for the intervention in a focus group study. This will be followed by designing and evaluating the effectiveness of the intervention in a randomised controlled trial.

## Data

The dataset relied on in this article is available from the corresponding author on reasonable request.

## Trial registration number

This study is part of a larger study aiming to develop and evaluate a multi-component intervention for improving personal continuity in general practice (Netherlands Trial Register: Trial NL8132)
